# *De novo* Genome Assembly of the *indica* Rice Variety IR64 Using Linked-Read Sequencing and Nanopore Sequencing

**DOI:** 10.1534/g3.119.400871

**Published:** 2020-03-17

**Authors:** Tsuyoshi Tanaka, Ryo Nishijima, Shota Teramoto, Yuka Kitomi, Takeshi Hayashi, Yusaku Uga, Taiji Kawakatsu

**Affiliations:** * Division of Basic Research, Institute of Crop Science, National Agriculture and Food Research Organization, Tsukuba, Ibaraki 305-8518, Japan; † Division of Biotechnology, Institute of Agrobiological Sciences, National Agriculture and Food Research Organization, Tsukuba, Ibaraki 305-8604, Japan

**Keywords:** *De novo* genome assembly, *indica* rice, IR64, linked-read sequencing, nanopore sequencing

## Abstract

IR64 is a rice variety with high-yield that has been widely cultivated around the world. IR64 has been replaced by modern varieties in most growing areas. Given that modern varieties are mostly progenies or relatives of IR64, genetic analysis of IR64 is valuable for rice functional genomics. However, chromosome-level genome sequences of IR64 have not been available previously. Here, we sequenced the IR64 genome using synthetic long reads obtained by linked-read sequencing and ultra-long reads obtained by nanopore sequencing. We integrated these data and generated the *de novo* assembly of the IR64 genome of 367 Mb, equivalent to 99% of the estimated size. Continuity of the IR64 genome assembly was improved compared with that of a publicly available IR64 genome assembly generated by short reads only. We annotated 41,458 protein-coding genes, including 657 IR64-specific genes, that are missing in other high-quality rice genome assemblies IRGSP-1.0 of *japonica* cultivar Nipponbare or R498 of *indica* cultivar Shuhui498. The IR64 genome assembly will serve as a genome resource for rice functional genomics as well as genomics-driven and/or molecular breeding.

IR64 is an iconic *indica* rice (*Oryza sativa* L.) variety that was developed by the International Rice Research Institute in the Philippines in 1985 (Mackill and Khush 2018). IR64 is a descendant of the “miracle rice” IR8, the initial variety of the Green Revolution. IR8 dramatically increased grain yield owing to the semi-dwarfing gene *sd1*. In addition to high yield, IR64 possesses high eating quality and disease resistance, therefore IR64 has been one of the most popular rice varieties grown in Southeast and South Asia from the late 1980s to the early 2000s. Modern varieties with higher yield and improved disease resistance have replaced IR64 in many countries over the past two decades. Importantly, those modern varieties are mostly progenies or relatives of IR64 (Mackill and Khush 2018). In addition, near-isogenic lines conferring novel and improved traits, such as drought tolerance and submergence resistance, have been developed in the IR64 genetic background. Therefore, genetic analysis of IR64 remains extremely important for further improvement of IR64 or its progenies.

The reference genome sequence of the rice *japonica* variety Nipponbare was analyzed by BAC-by-BAC sequencing using Sanger sequencing technology (Goff *et al.* 2002, IRGSP 2005). Advances in high-throughput sequencing technologies have enabled whole-genome resequencing of thousands of rice *japonica*, *indica*, and *aus* varieties, as well as more distantly related *Oryza* species. Reference-based resequencing is a powerful method to detect small polymorphisms used for quantitative trait loci analysis and genome-wide association study (Huang *et al.* 2010, Wang *et al.* 2018). However, resequencing is not applicable for large structural variations or highly diversified regions. Draft *de novo* genome assembly of IR64, generated by short reads, has been reported, but the assembly is highly fragmented and consists of thousands of scaffolds (Schatz *et al.* 2014). In 2014, chromosome-level genome sequences of the *indica* variety Shuhui498 (R498) were published (Du *et al.* 2017). This genome was determined by hybrid assembly using PacBio and Illumina platforms. The quality of the assembly was comparable to BAC-by-BAC sequences of the Nipponbare genome.

Synthetic long-read technologies enable virtual ultra-long reads to be derived from short reads generated by high-throughput sequencers and single-molecule sequencers generate ultra-long reads. The assemblies based on these long reads have higher contiguity than those based on short reads only. In the present study, we sequenced the IR64 genome using two platforms: 10x Genomics Chromium linked-reads and the single-molecule sequencer Oxford Nanopore MinION. We integrated linked-read sequencing data and nanopore sequencing data to construct the IR64 genome assembly (Figure 1). We used a publicly available genetic linkage map constructed from recombinant inbred lines (RILs) derived from IR64 and Azucena to construct chromosome-level superscaffolds. The quality of the IR64 genome assembly is comparable to that of the current rice reference genomes of *japonica* Nipponbare and *indica* Shuhui498, based on the completeness and accuracy of the genome sequences and comparative analysis of genes. Collectively, we provide a novel genome resource for the rice community and an additional option for the cost-effective *de novo* genome assembly approach.

**Figure 1:**
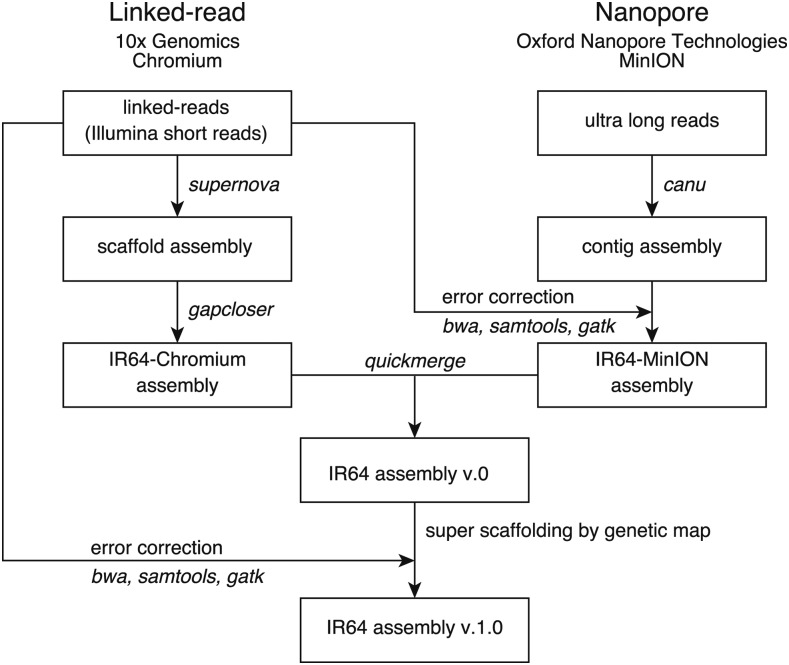
Schematic illustration of *de novo* assembly of the IR64 genome. Software used for analysis are indicated by italic.

## Materials and Methods

### Plant material and DNA extraction

The seeds of IR64 (International Rice Genebank Collection #66970, selfed for at least 10 times at National Institute of Agrobiological Sciences, Japan) were sterilized and incubated on Murashige and Skoog medium supplemented with 3% sucrose and 1% agar at pH 5.8 in a plant box at 28° for 8 days. Leaves from the 8-day-old seedlings were frozen in liquid nitrogen and ground to a fine powder with a mortar and pestle. High-molecular-weight DNA was extracted with Buffer G2 (Qiagen) supplemented with proteinase K and RNase A at 60 ° overnight with gentle agitation. After centrifugation at 2000 ×*g* for 30 min, the supernatant was loaded to a genomic-tip 100 (Qiagen) pre-equilibrated with Buffer QBT (Qiagen) and washed with Buffer QC (Qiagen) twice. DNA was eluted with Buffer QF (Qiagen), precipitated with isopropyl alcohol, washed with 70% ethanol, and dissolved in Buffer EB (Qiagen). The concentration of DNA was measured with the Qubit dsDNA High Sensitivity Assay Kit (Invitrogen).

### Public rice genome sequences and annotation data

Genome sequences and annotation data for *O. sativa* subsp. *japonica* Nipponbare (IRGSP-1.0) and *O. sativa* subsp. *indica* Shuhui498 (R498) were downloaded from the RAP-DB (https://rapdb.dna.affrc.go.jp/) (Kawahara *et al.* 2013; Sakai *et al.* 2013) and MBKBASE (http://www.mbkbase.org/R498/) (Du *et al.* 2017) databases, respectively. We also downloaded publicly available IR64 genome sequences from the Schatz Laboratory (http://schatzlab.cshl.edu/data/rice/) (Schatz *et al.* 2014). Given that no coding sequences (CDS) and protein sequences were accessible on the website, we extracted CDS sequences from the genome sequence using a GFF file and translated into protein sequences. For detection of repetitive elements, we used mipsREdat_9.3p_Poaceae_TEs.fasta downloaded from the PGSB database (http://pgsb.helmholtz-muenchen.de/plant/) (Spannagl *et al.* 2017).

### Linked-read sequencing

The linked-read library was prepared with the Chromium Genome Reagent Kit (10x Genomics) and sequenced on one lane of an Illumina HiSeq X platform at Macrogen Japan. The linked-reads were assembled using the Supernova v.2.0.1 assembler with default parameters, except for “–maxreads=142000000” to achieve 56× raw coverage, in accordance with the manufacturer’s instructions. The initial draft genome assembly IR64_Chromium was presented in pseudohaplotype format. Gap closing was conducted with GAPCLOSER v.1.12 for the further scaffolding (Luo *et al.* 2012). The resulting sequences were used for the further scaffolding.

### Nanopore sequencing

A DNA library for MinION sequencing was prepared based on the protocol for the Rapid Lambda Control Experiment using the Rapid Sequencing Kit (Oxford Nanopore Technologies). The library was loaded onto MinION R9.5 SpotON Flow Cells (Oxford Nanopore Technologies). Base calling was performed by MinKnow. Genome assembly was conducted using Canu v1.6 with the parameter “-nanpore-raw”. The assembled contigs were corrected using short reads obtained from the linked-read library using the HiSeq X platform. The paired-end reads were mapped to the assembled sequences by BWA-0.7.15 with the parameters “mem -M -T 30” (Li and Durbin 2009). Processing was performed using samtools-1.4 with the steps “view -q 30 -F 0x100” and “view -f 0x2” (Li 2011). Finally, polymorphisms detected by the Genome Analysis Toolkit HaplotypeCaller with the options “-out_mode EMIT_VARIANTS_ONLY–variant_index_type LINEAR–variant_index_parameter 128000–filter_reads_with_N_cigar” (McKenna *et al.* 2010) were incorporated into the assembled sequences using bcftools-1.4 with the option “consensus” (Li 2011).

### Construction of IR64 v. 1.0 assembly

Scaffolds from 10x Genomics Chromium and contigs from the Nanopore MinION platforms were integrated by Quickmerge (version 3) with the default settings (Chakraborty *et al.* 2016). Then, we mapped GBS data for the IR64 × Azucena RILs population downloaded from the Rice Diversity database (http://www.ricediversity.org/data/) (Spindel *et al.* 2013). A total of 30,984 single-nucleotide polymorphism (SNP) markers were mapped to the IR64 consensus sequences by BLASTN with the thresholds ≥90% of identity and coverage (Camacho *et al.* 2009). Based on the genetic distance, scaffolds were aligned along the chromosomal position. Given that not all markers were aligned consistent with the genetic distance, we discarded orphan SNP markers and markers with inconsistent positions within 1000 bp. Next, 599 possible erroneous assemblies were manually curated. We split the erroneous assembly at the gaps between contigs and moved them to the correct loci, according to genetic map. Finally, the curated sequences were corrected using short reads obtained from the linked-reads library generated with the HiSeq X platform as described above. The genome size of IR64 was estimated from the *k*-mer frequency distribution (Zhang *et al.* 2012) using JellyFish-2.2.10 (Marcias and Kingsford 2011) with a *k*-mer size of 25.

### Genome annotation

We annotated gene models using MAKER 2.31.10, which integrates a RNA sequencing (RNA-seq) based gene model, protein homology, and *ab initio* gene prediction. To construct RNA-seq-based gene models, we used the publicly available IR64 RNA-seq reads (Xiang *et al.* 2017). After adaptor and quality trimming using trimmomatic-0.30 (ILLUMINACLIP: TruSeq3-SE.fa:2:30:10 LEADING:15 TRAILING:15 SLIDINGWINDOW:4:15 MINLEN:32) (Bolger *et al.* 2014), the reads were mapped to the IR64 v.1.0 genome assembly using HISAT2 (version 2.0.5) (–min-intronlen 20–max-intronlen 10000–downstream-transcriptome-assembly–rna-strandness RF) (Kim *et al.* 2015) and gene structures were predicted by StringTie (version 1.3.3) with the default parameters (Pertea *et al.* 2015). Finally, all gene model sets were integrated into single RNA-seq-based gene model sets. For protein mapping, we used the IRGSP-1.0 and R498 gene models (Du *et al.* 2017; Kawahara *et al.* 2013). For *ab initio* gene prediction, we used SNAP (version 2006-07-28) (Korf 2004) and AUGUSTUS (version 3.3.1) (Stanke and Waack 2003). For functional annotation, we used InterProScan (version 5.2.4-63.0) (-f XML) (Jones *et al.* 2014). Domain information and gene ontology (GO) data were extracted from the results. Repetitive regions were detected by REPEATMASKER (v.4.0.7) using mipsREdat_9.3p_Poaceae_TEs.fasta and the default settings.

### Validation of IR64 v.1.0 sequence

We calculated the LTR assembly index (LAI) for the IR64 v. 1.0 assembly and IR64 CSHL scaffolds (Ou *et al.* 2018). To locate LTR retrotransposons (LTR-RTs), we used LTR-finders (Xu and Wang 2007). The parameters used for LAI followed recommendations in the LTR_retriever manual. For genome alignment, we used MUMMER (version 4.0.0beta2) (nucmer–maxgap = 5001–mincluster = 100, and delta-filter -q -g) (Marçais *et al.* 2018). We aligned the linked-read assembly, MinION assembly, scaffolds, and superscaffolds of the IR64 genome to the IRGSP-1.0 and R498 genome sequences. We mapped IRGSP-1.0 representative and predicted genes, and R498 annotated genes to the IR64 genome using GMAP (2017-03-17) (-f gff3_gene) with ≥95% identity and ≥90% coverage (Wu and Watanabe 2005). Genomic regions of unmapped IRGSP-1.0 representative genes were extracted with 1000 bp of the upstream and downstream flanking regions. The paired-end reads of IR64 were mapped using BWA-0.7.15 with the parameters “mem -M -T 30”. Mapped reads were processed by samtools-1.4 with the parameters “view -q 30 -F 0x100” and “view -f 0x2”. A read count for each site was performed using samtools-1.4 with the parameter “mpileup -u -v”. We also conducted a homology search of IR64 genes using BLASTP against our annotation data and the genome mapping using GMAP with the same aforementioned threshold. We downloaded BUSCO version 3.0.2 and the Liliopsida *odb10* dataset (https://busco.ezlab.org/) (Simão *et al.* 2015). We ran BUSCO.py with the default settings.

### Transcript comparison

We conducted a homology search of IR64 proteins against IRGSP representative genes, IRGSP predicted genes, and R498 genes using BLASTP with a threshold of E-value < 1E^−10^ (Camacho *et al.* 2009). IR64 genes that lacked homologies to other genes were mapped to the IR64 (Os-IR64-Draft-CSHL-1.0), IRGSP, and R498 genome sequences using GMAP (2017-03-17) (-f gff3_gene) with ≥95% identity and ≥90% coverage. Expression evidence of IR64 proteins was evaluated using the RNA-seq data. We mapped 16 single-end RNA-seq samples from either the root or the shoot using BWA-0.7.15 with the parameters “mem -M -T 30”. Processing and read counts for each site were performed using samtools-1.4 with the parameters “view -q 30 -F 0x100” and “mpileup -u -v”. Coverage by RNA-seq for each transcript was calculated.

### Data availability

The datasets generated during the current study are available in the Sequence Read Archive under accession number PRJD88810. The genome assembly of IR64 v.1.0 is available under the DDBJ assembly accession numbers BLLQ01000001-BLLQ01000012. All genomic data analyzed in this study can be downloaded and visualized in the ROOTomics Database (https://rootomics.dna.affrc.go.jp/en/research/IR64), hosting JBrowse for visualizing genome annotation data and BLAST server. Supplemental files below are available at FigShare. Table S1. Annotation data of possible missing genes in IR64 genome. Figure S1. Genome alignment between IRGSP-1.0 genome and IR64 scaffolds. Red and blue dots represent forward and reverse alignments, respectively. Figure S2. Chromosome alignments between R498 and IR64 v.1.0. Figure S3. Chromosome alignments between IRGSP-1.0 and IR64 v.1.0. Figure S4. Chromosome alignments of chromosome 6 from 13 Mbp to 19 Mbp. Figure S5. Chromosomal distribution of genes mapped on IR64 v.1.0 unanchored sequences. Figure S6. Fraction of missing genic regions in IR64 covered by paired-end reads obtained from linked-read sequencing of IR64 genome. Figure S7. Distribution of gene ontologies. Supplemental material available at figshare: https://doi.org/10.25387/g3.10058657.

## Results and Discussion

### de novo assembly of IR64 genome sequence

**Figure 2:**
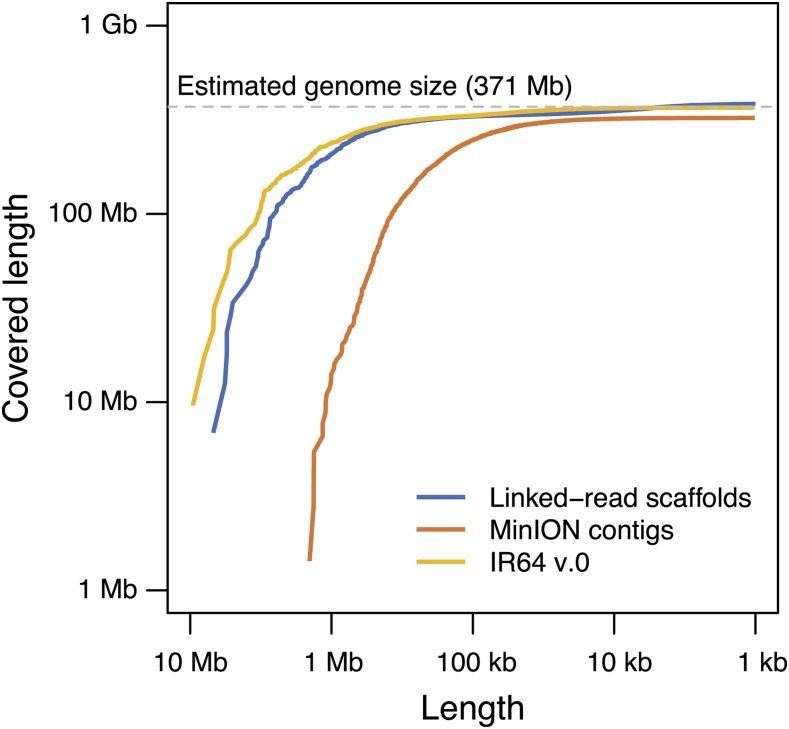
Distribution of assembled sequence length. The x-axis represents sequence length of scaffolds/contigs and the y-axis represents covered length of genome. Linked-read scaffolds (blue), MinION contigs (orange) and merged scaffolds (yellow) are shown.

We sequenced the IR64 genome using linked-read sequencing and obtained 910 million raw reads, equivalent to 138 Gb (∼368×) (Table 1). The draft genome assembly based on 142 million linked-reads (∼56×), IR64-Chromium, consisted of 10,153 scaffolds with a total sequence length of 384 Mb. The maximum length and N50 of IR64-Chromium scaffolds were 6.9 and 1.2 Mb, respectively. We also sequenced the IR64 genome using nanopore sequencing and obtained 1.4 million raw reads with an average length of 1.45 kb, equivalent to 9.3 Gb (∼24×) (Table 1). The draft genome assembly based on nanopore sequencing, IR64-MinION, consisted of 3,258 contigs with a total sequence length of 323 Mb. The maximum length and N50 of IR64-MinION contigs were 1.4 Mb and 224 kb, respectively. The distribution of assembled sequence lengths differed between IR64-Chromium and IR64-MinION (Figure 2). In IR64-Chromium, over 80% of the scaffolds were shorter than 10 kb. However, 86% of genome sequences were covered by 4% of the scaffolds, which were longer than 100 kb. In contrast, the fractions of short contigs (<10 kb) and long contigs (100 kb) were 17% and 30%, respectively, in IR64-MinION.

**Table 1:** Summary statistics for the linked-reads and MinION data

	Linked-Reads	MinION
Number of reads	910,295,956	1,449,788
Total data size (bp)	137,454,689,356	9,276,893,086
Read depth (x)	368	24

Genome size was 373 Mb based on IRGSP-1.0.

Next, we merged IR64-Chromium and IR64-MinION to construct a longer assembly. Before this, we polished the IR64-MinION contig sequences using high-quality short reads obtained by linked-read sequencing. We identified and corrected 82,829 SNPs (20.9%), 285,503 insertions (72.0%), 24,981 deletions (6.3%), and 3,404 multiple polymorphisms (0.9%). We integrated IR64-Chromium and IR64-MinION into the IR64 v.0 draft assembly to improve the assembly contiguity. IR64 v.0 consisted of 1770 scaffolds, for which the maximum length and N50 were 9.6 Mb and 1.6 Mb, respectively (Table 2). Although distribution of the scaffold lengths of IR64 v.0 was intermediate between those of IR64-Chromium and IR64-MinION, 96 extremely long scaffolds (>1 Mb) covered 238 Mb (64.2% of the estimated genome size, 371 Mb). These results clearly demonstrate that integration of linked-read sequencing and nanopore sequencing is a promising *de novo* assembly strategy.

**Table 2:** Summary statistics for the genome assemblies

Platforms	Linked-Read	MinION	IR64 v.0	IR64 v.1.0
Number of sequences	10,153	3,258	1,770	13
Total length (bp)	384,086,199	323,606,076	367,012,357	367,109,233
N50 (bp)	1,187,152	224,507	1,646,684	27,827,038
Minimum length (bp)	1,000	1,011	1,011	
Maximum length (bp)	6,875,104	1,431,035	9,584,587	
Number of Ns (bp)	22,492,160	0	19,621,636	19,672,336
GC content wo Ns (%)				42.8

To obtain the chromosome-scale assembly, we aligned the scaffolds by mapping the genetic markers constructed from IR64 × Azucena RILs (Spindel *et al.* 2013). Anchored and ordered 477 scaffolds covered 337 Mb (91.8% of the total scaffolds length). The length of 1293 unanchored scaffolds ranged from 1 kb to 380 kb. A total of 26,257 SNP markers, mapped to IR64 v.0 scaffolds, were clustered into 2803 physical regions. After filtering out less reliable regions (only one SNP marker was mapped) and small regions (<1 kb), 592 regions with 23,851 SNP markers and 333 Mb in length remained. From the manual check of 599 possible erroneous regions, we observed that 84 scaffolds were separated in different genomic regions (Figure S1) and 25 regions of the scaffolds were nested, where contigs located at different genomic regions were inserted. We manually corrected these misassemblies, according to the genetic map. To elucidate the origins of the chimeric scaffolds, we mapped IR64-Chromium scaffolds, IR64-MinION contigs, and IR64 v.0 scaffolds to IRGSP-1.0 using MUMMER. The frequencies of chimeric sequences mapped to the different IRGSP-1.0 chromosomes were 3.2% for IR64-Chromium, 23.5% for IR64-MinION, and 9.6% for IR64 v.0. Therefore, IR64-MinION was a major source of chimeric assemblies. After the manual curation, we constructed IR64 pseudomolecules with 12 chromosomes and one concatenated sequence of unanchored scaffolds.

Finally, we corrected sequence errors with Illumina reads and constructed IR64 v.1.0. IR64 v.1.0 was 367 Mb in total and 19.7 Mb of ambiguous nucleotide stretches (Ns) (Table 2), covering 98.9% of the estimated IR64 genome size.

### Validation of IR64 v.1.0

**Figure 3:**
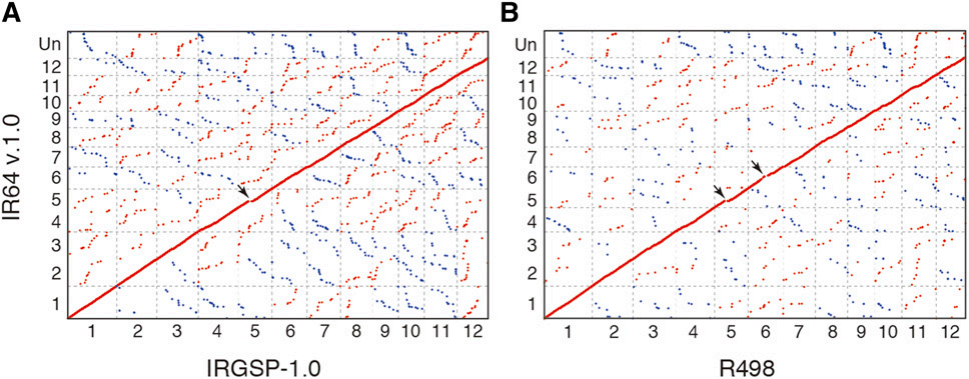
Genome alignment between the IR64 v.1.0 assembly and other rice reference genomes. Genome alignments were constructed using MUMMER4 A) between IRGSP-1.0 (japonica) and IR64 (indica), and B) between R498 (indica) and IR64 (indica). Numerals indicate chromosome number. Un indicate concatenated unanchored sequences. Red and blue dots represent forward and complement alignments, respectively. Arrows indicate the alignment gaps significantly larger than the resolution of IR64 v.1.0.

While IR64 v.1.0 covered 98.9% of the estimated genome size of IR64, there were 19.7 Mb of ambiguous nucleotides and 34.4 Mb unanchored sequences. The GC content of IR64 v.1.0 (42.8%) was slightly lower than those of IRGSP-1.0 (43.6%) and R498 (43.6%), possibly because of unassembled regions in IR64 v.1.0. Genome alignment of IR64 against IRGSP-1.0 and R498 showed highly conserved genome structures (Figure 3). A higher number of inter-chromosome alignments were observed between IRGSP-1.0 and IR64 v.1.0 than between R498 and IR64 v.1.0, likely reflecting the sequence diversity depending on the evolutionary distance. Unanchored sequences were distributed on all chromosomes of IRGSP-1.0 and R498, even though the aligned regions of unanchored sequences were distinct (Figure 3). We detected the alignment gaps, which were significantly larger than the resolution of IR64 v.1.0, in chromosomes 5 and 6 (Figure 3). The gap in chromosome 5 was observed both between IR64 and IRGSP-1.0, and between IR64 and R498, whereas that in chromosome 6 was only observed between IR64 and R498 (Figure S2 and S3). Closer inspection revealed the gap in chromosome 6 was caused by a large inversion between IR64 and R498 (Figure S4), previously reported between IRGSP-1.0 and R498 (Du *et al.* 2017). The inversion does not occur between IR64 and IRGSP-1.0 (Figure S4). These results suggest that this inversion is specific to R498, but is not a signature of *indica* rice varieties. This highlights the power of *de novo* genome assembly and the importance of multiple *indica* reference genomes.

**Figure 4:**
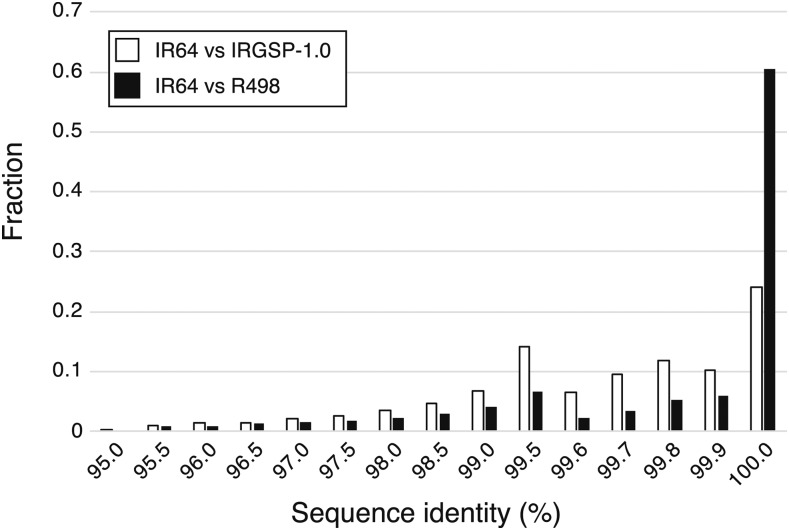
Distribution of sequence similarity of genes between IR64 and R498 (black) and IRGSP-1.0 representative genes (white). Sequence alignment between transcripts (IRGSP-1.0 or R498) and genomes (IR64) and calculation of identity were performed using GMAP.

To analyze the genome sequences associated with biological functions, we mapped the IRGSP-1.0 and R498 genes against IR64 v.1.0. We observed that 89.7% of IRGSP-1.0 representative genes, 71.5% of IRGSP-1.0 predicted genes, and 92.7% of R498 genes were mapped to IR64 v.1.0. These results suggested that genic regions were highly conserved among rice varieties and could be well assembled, whereas predicted genes without functional evidence were less conserved or assembled. In addition, sequence similarities between R498 genes and IR64 v.1.0 were higher than those between IRGSP-1.0 genes and IR64 v.1.0, which reflected the evolutionary relationship (Figure 4).

We next mapped 1,584 IRGSP-1.0 representative genes, located on all chromosomes of IRGSP-1.0, on the unanchored sequences of IR64 v.1.0. Genome-wide distribution of R498 genes mapped on unanchored sequences of IR64 v.1.0 was also observed. IRGSP-1.0 representative genes and R498 genes, mapped on unanchored sequences of IR64 v.1.0, were enriched within the gap in chromosome 5 described above (one-tailed Fisher exact test: p-value = 3.1e-91 for IRGSP-1.0 and p-value = 4.5e-319 for R498; Figure S5). This suggests that the alignment gap in the middle of chromosome 5 was caused by incorporation of this region into unanchored sequences in IR64 v.1.0, possibly due to fragmented assembly and/or low marker density. We determined that 1,081 IRGSP-1.0 representative genes were missing in IR64 v.1.0. To validate the potential gene loss in IR64 v.1.0 due to misassembly, we mapped raw short reads from the linked-read library of IR64 to the genomic regions of these 1,081 IRGSP-1.0 genes (transcribed regions and 1 kb upstream/downstream flanking regions; total length 4.17 Mb). We observed that 843 kb were not covered by short reads and 27 regions were larger than 5 kb. These long deletions were distributed on all chromosomes except chromosomes 5, 8, and 10. On the other hand, we observed that 3.3 Mb (∼80% of missing sequences in IR64 v.1.0) were covered by at least 10 short reads (Figure S6), suggesting that these missing sequences in IR64 v.1.0 exist in the IR64 genome, but were not assembled by linked-reads or MinION data. We showed that 179 IRGSP-1.0 representative genes were located in the genomic regions not covered by IR64 short-read data (Table S1). To focus on non-genic regions, we used the LAI to evaluate assembly continuity using LTR-RTs (Ou *et al.* 2018). We compared the quality of IR64 v.1.0 and that of previously published IR64 scaffolds using the LAI. The LAIs of IR64 v.1.0 and Os-IR64-Draft-CSHL-1.0 were 8.69 and 7.68, respectively. This result supported our conclusion that the IR64 v.1.0 assembly showed better quality in terms of chromosome-level assembly than that of the public IR64 scaffolds.

### Gene annotation and repeat analysis

We annotated 41,458 IR64 protein-coding genes using 15 RNA-seq datasets (Xiang *et al.* 2017), rice annotated genes from IRGSP-1.0 and R498 combined with *ab initio* gene predictions. We observed that 32,341 (78%) of IR64 v.1.0 genes were expressed at least in the root or shoot. BUSCO analysis detected 92.3% and 1.5% of the genes as single-copy and duplicated, respectively, suggesting that the IR64 v.1.0 gene set covers almost all IR64 genes in the genome. We assigned functional domain information for 27,922 transcripts using InterProScan, while information for 13,536 transcripts was unknown. In addition, we assigned GO terms, based on the InterPro domain information, to 19,000 transcripts (Figure S7). The distribution of GO terms of predicted IR64 v.1.0 genes was comparable to those of IRGSP-1.0 and R498 genes, while exact numbers of assigned GO terms differed owing to the distinct number of predicted genes.

Next, we compared the IR64 v.1.0 gene set with the IRGSP-1.0 and R498 gene sets (Table 3). We observed that a higher number of IR64 v.1.0 genes were missing in the IRGSP-1.0 gene set than in the R498 gene set, presumably reflecting the evolutionary distance between *japonica* and *indica* rice, rather than the quality of genome assembly and gene annotation. We detected 2,649 genes as candidates of IR64 v.1.0 specific genes, which are missing in either the IRGSP-1.0 or R498 gene sets. These genes were distributed on all IR64 chromosomes even in an unanchored sequence. To examine whether these IR64 genes were simply unannotated in the IRGSP-1.0 and R498 genomes, we mapped IR64 genes to the latter two genomes. We observed that 657 genes were not mapped on both genomes. Therefore, these genes were candidates for IR64-specific genes. By mapping these genes to public IR64 scaffolds, we determined that 550 genes were newly assigned in the IR64 genome. In addition, 1531 of 2649 candidates for IR64-specific genes were expressed. Among the 550 genes that were newly assigned in the IR64 genome and only detected in the IR64 genome, 378 genes were expressed in either the root or shoot.

**Table 3:** Number of IR64 v.1.0 genes that are missing in IRGSP-1.0 and R498 for individual chromosomes

	BOTH	IRGSP-1.0	R498
chr01	249	422	148
chr02	215	398	154
chr03	206	318	113
chr04	203	439	106
chr05	162	265	112
chr06	182	356	125
chr07	145	364	87
chr08	165	373	102
chr09	156	353	82
chr10	164	290	93
chr11	180	365	96
chr12	144	342	102
chrUn	478	490	95
Total	2,649	4,775	1,415

Finally, we compared our genes with the Os-IR64-Draft-CSHL-1.0 annotation, based on the Illumina reads assembly by ALLPATH-LG (Schatz *et al.* 2014). BUSCO analysis of the Os-IR64-Draft-CSHL-1.0 annotation assigned 95.5% of the genes as single-copy genes. This percentage was higher than that of our annotation data. However, 664 genes (20.3%) were duplicated. These results suggested that the public scaffolds still contained artificially duplicated genome sequences. In addition, 41,773 of 51,597 genes (80.9%) were observed in our annotation data, and approximately one-third (34.7%) of no-hit genes were less than 300 bp (100 amino acids), which suggested that these genes might represent erroneous gene structures.

**Figure 5:**
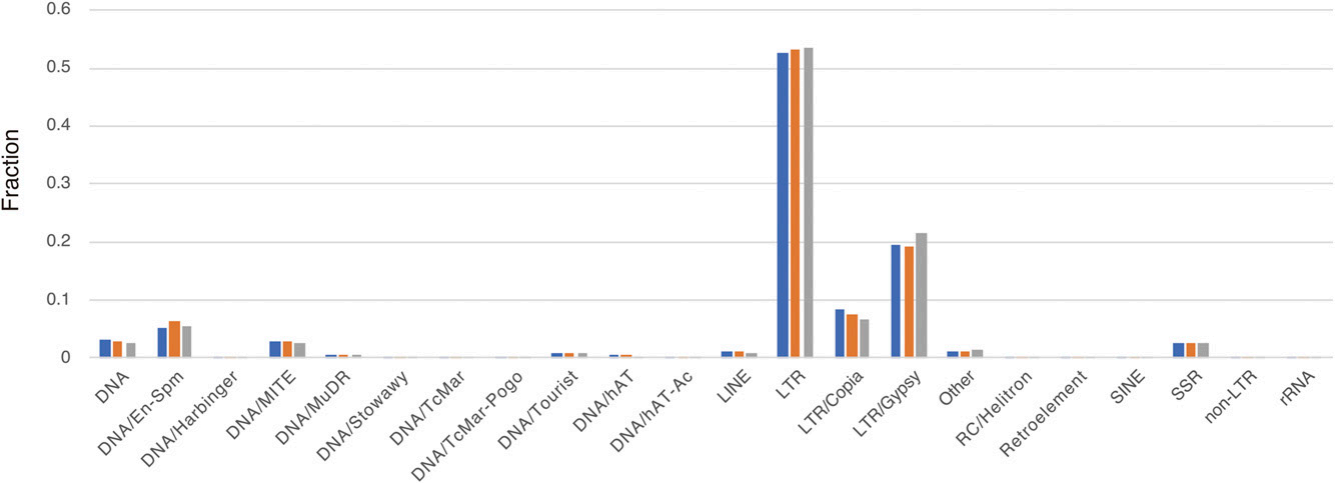
Distribution of repeat elements in IR64 (blue), IRGSP-1.0 (orange), and R498 (gray). Repeat elements were calculated from the results of RepeatMasker.

From the repeat masking, we detected 692,604 repetitive regions that contained 425,373 LTRs, 126,414 DNA transposons, and 116,026 simple sequence repeats, including centromeric and telomeric sequences. Among these elements, LTR/Gypsy (64,041: 40.2 Mbp, 11.0% of the genome), DNA/MITE (38,316: 189 Kbp, 0.1%), and LTR/Copia (35,667: 17.5 Mbp, 4.8%) were the three most abundant repeat elements (Figure 5). The distributions of repeat elements were similar among the three genomes, suggesting that the present hybrid assembly showed comparable performance for repetitive regions as the PacBio-based assembly and BAC-by-BAC Sanger sequencing-based assembly.

## Conclusions

This is the first report describing the *de novo* assembly using integration of linked-read sequencing and nanopore sequencing in plant genomes. Given that the 10x Genomics Chromium platform was developed and optimized for the human genome, its performance for a plant genome was not identical to the human case. The present results showed that *de novo* assembly only by linked-reads resulted in more than 10,000 fragments of genome sequences. These data were much worse than the public data (Schatz *et al.* 2014). In the case of MinION data, the number of contigs and the total length (323 Mb) were fewer than those of the public data (345 Mb in 2919 scaffolds). The integration of these two assemblies resulted in construction of a much improved assembly (367 Mb in 1770 scaffolds). Moreover, using publicly available genetic map information, we could construct chromosome-level superscaffolds. Assembly quality was more improved than the public data, on the basis of the LAI and BUSCO analysis. Even if library preparation and sequencing had been conducted by a commercial provider, the total cost would have been less than 9000 USD (4500 USD each for linked-read sequencing and nanopore sequencing) in Japan. Recent trends for *de novo* assembly performed on non-model organisms, and even cultivars, demands a cheaper but effective analysis scheme for high-throughput sequencing analysis. The present study shows that *de novo* genome assembly using different long-reads platforms provides promising results.

## References

[bib1] Bolger A. M., Lohse M., and Usadel B., 2014 Trimmomatic: a flexible trimmer for Illumina sequence data. Bioinformatics 30: 2114–2120. 10.1093/bioinformatics/btu170 24695404 PMC4103590

[bib2] Camacho C., Coulouris G., Avagyan V., Ma N., Papadopoulos J., 2009 BLAST+: architecture and applications. BMC Bioinformatics 10: 421 10.1186/1471-2105-10-421 20003500 PMC2803857

[bib3] Chakraborty M., Baldwin-Brown J. G., Long A. D., and Emerson J. J., 2016 Contiguous and accurate *de novo* assembly of metazoan genomes with modest long read coverage. Nucleic Acids Res. 44: e147. 27458204 10.1093/nar/gkw654 PMC5100563

[bib4] Du H., Yu Y., Ma Y., Gao Q., Cao Y., 2017 Sequencing and *de novo* assembly of a near complete *indica* rice genome. Nat. Commun. 8: 15324 10.1038/ncomms15324 28469237 PMC5418594

[bib5] Goff S. A., Ricke D., Lan T.-H., Presting G., Wang R., 2002 A Draft Sequence of the Rice Genome (*Oryza sativa* L. ssp. *japonica*). Science 296: 92–100. 10.1126/science.1068275 11935018

[bib6] Huang X., Wei X., Sang T., Zhaong Q., Feng Q., 2010 Genome-wide association studies of 14 agronomic traits in rice landraces. Nat. Genet. 42: 961–967. 10.1038/ng.695 20972439

[bib7] International Rice Genome Sequencing Project, 2005 The Map-Based Sequence of the Rice Genome. Nature 436: 793–800. 10.1038/nature03895 16100779

[bib8] Kawahara Y., de la Bastide M., Hamilton J. P., Kanamori H., McCombie W. R., 2013 Improvement of the *Oryza sativa* Nipponbare reference genome using next generation sequence and optical map data. Rice (N. Y.) 6: 4 10.1186/1939-8433-6-4 24280374 PMC5395016

[bib9] Kim D., Langmead B., and Salzberg S. L., 2015 HISAT: a fast spliced aligner with low memory requirements. Nat. Methods 12: 357–360. 10.1038/nmeth.3317 25751142 PMC4655817

[bib10] Korf I., 2004 Gene finding in novel genomes. BMC Bioinformatics 5: 59 10.1186/1471-2105-5-59 15144565 PMC421630

[bib11] Li H., 2011 A statistical framework for SNP calling, mutation discovery, association mapping and population genetical parameter estimation from sequencing data. Bioinformatics 27: 2987–2993. 10.1093/bioinformatics/btr509 21903627 PMC3198575

[bib12] Li H., and Durbin R., 2009 Fast and accurate short read alignment with Burrows-Wheeler Transform. Bioinformatics 25: 1754–1760. 10.1093/bioinformatics/btp324 19451168 PMC2705234

[bib13] Luo R., Liu B., Xie Y., Li Z., Huang W., 2012 SOAPdenovo2: an empirically improved memory-efficient short-read *de novo* assembler. Gigascience 1: 18 10.1186/2047-217X-1-18 23587118 PMC3626529

[bib14] Mackill J. D., and Khush G. S., 2018 IR64: a high-quality and high-yielding mega variety. Rice (N. Y.) 11: 18 10.1186/s12284-018-0208-3 29629479 PMC5890005

[bib15] Marçais G., Delcher A. L., Phillippy A. M., Coston R., Salzberg S. L., 2018 MUMmer4: A fast and versatile genome alignment system. PLOS Comput. Biol. 14: e1005944 10.1371/journal.pcbi.1005944 29373581 PMC5802927

[bib33] Marçais G., and Kingsford C., 2011 A fast, lock-free approach for efficient parallel counting of occurrences of k-mers. Bioinformatics 27: 764–770. 10.1093/bioinformatics/btr011 PMC3051319 21217122

[bib16] McKenna A., Hanna M., Banks E., Sivachenko A., Cibulskis K., 2010 The Genome Analysis Toolkit: A MapReduce framework for analyzing next-generation DNA sequencing data. Genome Res. 20: 1297–1303. 10.1101/gr.107524.110 20644199 PMC2928508

[bib17] Ou, Shujun, J. Chen, N. Jiang 2018 Assessing genome assembly quality using the LTR Assembly Index (LAI). Nucleic Acids Res. 46: e126. 10.1093/nar/gky730 PMC6265445 30107434

[bib18] Pertea M., Pertea G. M., Antonescu C. M., Chang T. C., Mendell J. T., 2015 String Tie enables improved reconstruction of a transcriptome from RNA-seq reads. Nat. Biotechnol. 33: 290–295. 10.1038/nbt.3122 25690850 PMC4643835

[bib19] Jones P., Binns D., Chang H.-Y., Fraser M., Li W., 2014 InterProScan 5: genome-scale protein function classification. Bioinformatics 30: 1236–1240. 10.1093/bioinformatics/btu031 24451626 PMC3998142

[bib20] Sakai H., Lee S. S., Tanaka T., Numa H., Kim J., 2013 Rice Annotation Project Database (RAP-DB): an integrative and interactive database for rice genomics. Plant Cell Physiol. 54: e6 10.1093/pcp/pcs183 23299411 PMC3583025

[bib21] Schatz M. C., Maron L. G., Stein J. C., Wences A. H., Gurtowski J., 2014 Whole genome *de novo* assemblies of three divergent strains of rice (*O. sativa*) documents novel gene space of *aus* and *indica*. Genome Biol. 15: 506. 25468217 10.1186/s13059-014-0506-z PMC4268812

[bib22] Simão A. F., Waterhouse R. M., Ioannidis P., Kriventseva E. V., and Zdobnov E. M., 2015 BUSCO: assessing genome assembly and annotation completeness with single-copy orthologs. Bioinformatics 31: 3210–3212. 10.1093/bioinformatics/btv351 26059717

[bib23] Spannagl M., Nussbaumer T., Bader K. C., Miartis M. M., Seidel M., 2016 PGSB PlantsDB: updates to the database framework for comparative plant genome research. Nucleic Acids Res. 44: D1141–D1147. 10.1093/nar/gkv1130 26527721 PMC4702821

[bib24] Spindel J. E., Wright M., Chen C., Cobb J., Gage J., 2013 Bridging the genotyping gap: using genotyping by sequencing (GBS) to add high-density SNP markers and new value to traditional bi-parental mapping and breeding populations. Theor. Appl. Genet. 126: 2699–2716. 10.1007/s00122-013-2166-x 23918062

[bib25] Stanke M., and Waack S., 2003 Gene prediction with a hidden Markov model and a new intron submodel. Bioinformatics (Suppl 2) 19: ii215–ii225. 14534192 10.1093/bioinformatics/btg1080

[bib26] Wang W., Mauleon R., Hu Z., Chebotarov D., Tai S., 2018 Genomic variation in 3,010 diverse accessions of Asian cultivated rice. Nature 557: 43–49. 10.1038/s41586-018-0063-9 29695866 PMC6784863

[bib27] Weisenfeld N. I., Kumar V., Shah P., Church D. M., and Jaffe D. B., 2017 Direct determination of diploid genome sequences. Genome Res. 27: 757–767. 10.1101/gr.214874.116 28381613 PMC5411770

[bib28] Wu T. D., and Watanabe C. K., 2005 GMAP: a genomic mapping and alignment program for mRNA and EST sequences. Bioinformatics 21: 1859–1875. 10.1093/bioinformatics/bti310 15728110

[bib29] Xiang J., Wu H., Zhang Y., Zhang Y., Wang Y., 2017 Transcriptomic Analysis of Gibberellin- and Paclobutrazol-Treated Rice Seedlings under Submergence. Int. J. Mol. Sci. 18: 2225 10.3390/ijms18102225 PMC5666904 29064391

[bib30] Xu, Z. and H. Wang, 2007 LTR_FINDER: an efficient tool for the prediction of full-length LTR retrotransposons. Nucleic Acids Res. 35(Web Server issue): W265–W268. 10.1093/nar/gkm286 PMC1933203 17485477

[bib32] Zhang G., Fang X., Guo X., Li L., Luo R., 2012 The oyster genome reveals stress adaptation and complexity of shell formation. Nature 490: 49–54. 10.1038/nature11413 22992520

